# Structural Evolution of Delta (B.1.617.2) and Omicron (BA.1) Spike Glycoproteins

**DOI:** 10.3390/ijms23158680

**Published:** 2022-08-04

**Authors:** Ingrid Guarnetti Prandi, Carla Mavian, Emanuela Giombini, Cesare E. M. Gruber, Daniele Pietrucci, Stefano Borocci, Nabil Abid, Andrea R. Beccari, Carmine Talarico, Giovanni Chillemi

**Affiliations:** 1 Department for Innovation in Biological, Agro-Food and Forest Systems—DIBAF, University of Tuscia, Via S. Camillo de Lellis s.n.c., 01100 Viterbo, Italy; 2 Emerging Pathogen Institute, University of Florida, Gainesville, FL 32608, USA; 3 Department of Pathology, Immunology and Laboratory Medicine, University of Florida, Gainesville, FL 32610, USA; 4 Laboratory of Virology, INMI Lazzaro Spallanzani IRCCS, Via Portuense 292, 00149 Roma, Italy; 5 Institute of Biomembranes, Bioenergetics and Molecular Biotechnologies—IBIOM, CNR, 70126 Bari, Italy; 6 Institute for Biological Systems—ISB, CNR, Area della Ricerca di Roma 1, SP35d 9, 00010 Montelibretti, Italy; 7 Laboratory of Transmissible Diseases and Biological Active Substances LR99ES27, Faculty of Pharmacy, University of Monastir, Rue Ibn Sina, Monastir 5000, Tunisia; 8 High Institute of Biotechnology of Monastir, Department of Molecular and Cellular Biology, University of Monastir, Monastir 5000, Tunisia; 9 Dompé Farmaceutici SpA, Via Campo di Pile, 67100 L’Aquila, Italy

**Keywords:** SARS-CoV-2, COVID-19, spike, variants, molecular dynamics, omicron, delta

## Abstract

The vast amount of epidemiologic and genomic data that were gathered as a global response to the COVID-19 pandemic that was caused by SARS-CoV-2 offer a unique opportunity to shed light on the structural evolution of coronaviruses and in particular on the spike (S) glycoprotein, which mediates virus entry into the host cell by binding to the human ACE2 receptor. Herein, we carry out an investigation into the dynamic properties of the S glycoprotein, focusing on the much more transmissible Delta and Omicron variants. Notwithstanding the great number of mutations that have accumulated, particularly in the Omicron S glycoprotein, our data clearly showed the conservation of some structural and dynamic elements, such as the global motion of the receptor binding domain (RBD). However, our studies also revealed structural and dynamic alterations that were concentrated in the aa 627–635 region, on a small region of the receptor binding motif (aa 483–485), and the so-called “fusion-peptide proximal region”. In particular, these last two S regions are known to be involved in the human receptor ACE2 recognition and membrane fusion. Our structural evidence, therefore, is likely involved in the observed different transmissibility of these S mutants. Finally, we highlighted the role of glycans in the increased RBD flexibility of the monomer in the up conformation of Omicron.

## 1. Introduction

In 2020, an infection outbreak began in China that was responsible for human respiratory symptoms and was caused by a so far unknown viral strain, named Severe Acute Respiratory Syndrome Coronavirus 2 (SARS-CoV-2) [1,2,3].The disease spread rapidly in public health to the global level, causing severe disease and mortality [4].

SARS-CoV-2 is a member of the *Coronaviridae* family, a group of enveloped, positive-sense, single-stranded RNA (+ssRNA) viruses that includes another two viruses that are known to cause high pathogenic zoonotic infections: SARS-CoV-1 (detected in 2002–2003), which belongs to the same species of SARS-CoV-2 and Middle-East respiratory syndrome coronavirus (MERS-CoV), that was first isolated in 2012 and that is associated only occasionally to human–human transmission events [5]. The genome of SARS-CoV-2 is approximately 30 kb long, and it encodes for 4 structural proteins SP, 16 non-structural proteins NSP, and 9 accessory proteins [6].

Since the first isolate (MN908947.3), that was collected from COVID-19 patients in Hubei province of China in December 2019, SARS-CoV-2 has been evolving at a mutation rate estimated by 1.1 × 10^−3^ substitutions per site per year, corresponding to one substitution every 11 days [7]. Over two years of the pandemic, a number of substitutions have been fixed in the viral population, generating new variants or lineages that have been associated with an increase in transmissibility and/or with greater disease severity [8]. SARS-CoV-2 variants that are associated with a possible global public health risk are classified as “Variants Under Monitoring” (VUM), “Variants of Interest” (VOI), and “Variants of Concern” (VOC) by the World Health Organization (WHO) [4] and the Centers for Disease Control and Prevention (CDC) [9]. VUM are defined as variants with nucleotide mutations that can be associated with possible increased risk, but with unclear evidence of the epidemiological or phenotypic impact. VOIs are defined as variants with mutations that are associated with a potential increase in transmissibility, disease severity, immune escape, and diagnostic or therapeutic escape. VOCs are defined as variants with an increase in transmissibility or virulence, that are associated with more severe disease, a significant reduction in neutralization by antibodies, reduced effectiveness of treatments or vaccines, or diagnostic detection failures. All VOCs that have been described so far are characterized by mutations that cause conformational variations in the spike (S) glycoprotein regions that are involved in interactions with host cell surface receptors [4]. However, as more variants emerge and replace prior ones, VOC and VOI classification periodically evolves and updates, with the aim of sharpening surveillance and research targets [4]. For example, in 2021, wild-type (WT) SARS-CoV-2 lineages and Gamma variant VOC co-circulated at a low prevalence simultaneously with the then predominant Alpha variant VOC, in Italy [10,11] as well as elsewhere [12,13]. As the new Delta variant VOC, emerged in India in 2020 [14], the superseded Gamma and Alpha VOCs [15] were downgraded to VOIs [9].Another variant, Omicron, first reported in South Africa and Botswana in early November 2021 [16], and all its subvariants are the main VOCs that are causing infections in Italy [15] as well as worldwide [13]. Alpha, Gamma, and Delta have been re-classified as VUMs, and no VOIs have been designated [9].

Since its first identification, multiple vaccines that are based on different technologies and several therapeutics against SARS-CoV-2 and its associated COVID-19 disease have been successfully developed in a concerted global effort to provide effective protection against severe illness [17,18,19,20]. However, new emerging variants have acquired mutations that hampered drug and vaccine efficacy, with a progressive increase in breakthrough infections have been reported [21,22,23]. Therefore, obtaining a rapid and refined tri-dimensional structure analysis of the proteins that are mainly involved in the host-pathogen interaction can ensure that new variants with potential impact on public health are assessed in a timely manner. In particular, the study of protein dynamics that are involved in host-pathogen interaction processes can be useful to evaluate pathogenicity and transmission. Herein, we carry out an investigation into the S glycoprotein, starting with the comparison of the S glycoprotein of WT SARS-CoV-2 with those from beta-coronaviruses, that were previously isolated in bat and pangolin in China [24]; and followed by the comparison of WT SARS-CoV-2 and the Alpha variant [25]. In this work, we focus on the Omicron variant, which carries 50 mutations across the genome, including 30 in the S gene [13] and is widely spread, causing major concerns about its infectivity and immunological escape. This is in comparison to the previous widespread Delta variant, which harbored only 29 mutations, seven of which were in the S gene [26], as compared to the wild-type SARS-CoV-2.

## 2. Results

### 2.1. Altered Flexibility in the Delta and Omicron S Glycoprotein

**Figure 1:**
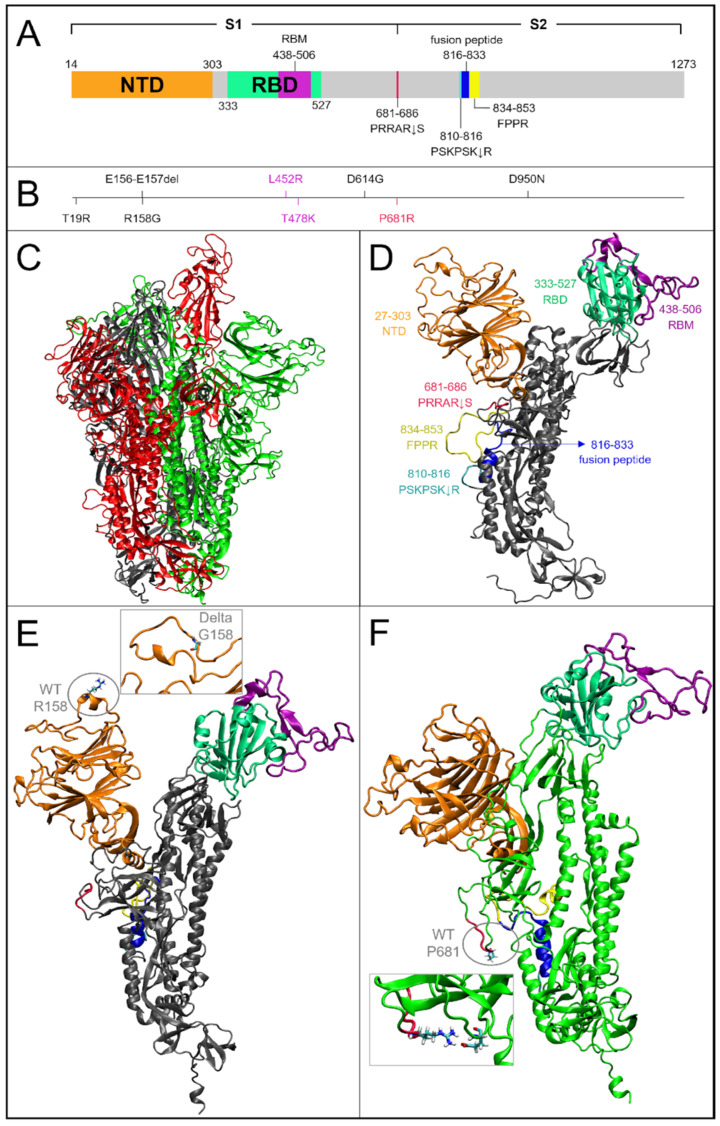
Domain organization and structural differences between the WT and Delta variant. (**A**) Graphical scheme of the spike domains from aa 14 to 1273: N-terminal domain (NTD) is represented in orange; the receptor-binding domain or RBD (aa 333–527) is highlighted in green; the receptor-binding motif, or RBM (aa 438–506), which is within RBD region, is represented in purple; the cleavage sequence at the S1/S2 boundary is colored in red; and the second cleavage site, the fusion peptide and the fusion peptide proximal region (FPPR) are represented in cyan, blue, and yellow, respectively. (**B**) Representation of Delta mutations, the numbering is aligned according to panel A. (**C**) Cartoon model visualization of the WT representative frame (629.8 ns of MD simulation) selected by cluster analyses. The monomers are colored in black, red (in up conformation), and green, respectively. (**D**) WT monomer 1 is represented with the same colors as the schematic domains in panel A. (**E**) Cartoon representation of WT monomer 1. The ellipse shows the conformation of the WT residue R158 and the rectangle shows the representative frame (655.4 ns) detail of the same region for the Delta variant with G158 mutation. (**F**) Cartoon representation of WT monomer 3. The ellipse and the rectangle show the conformations of the residue 681 for the WT and the Delta variant, respectively.

**Figure 2:**
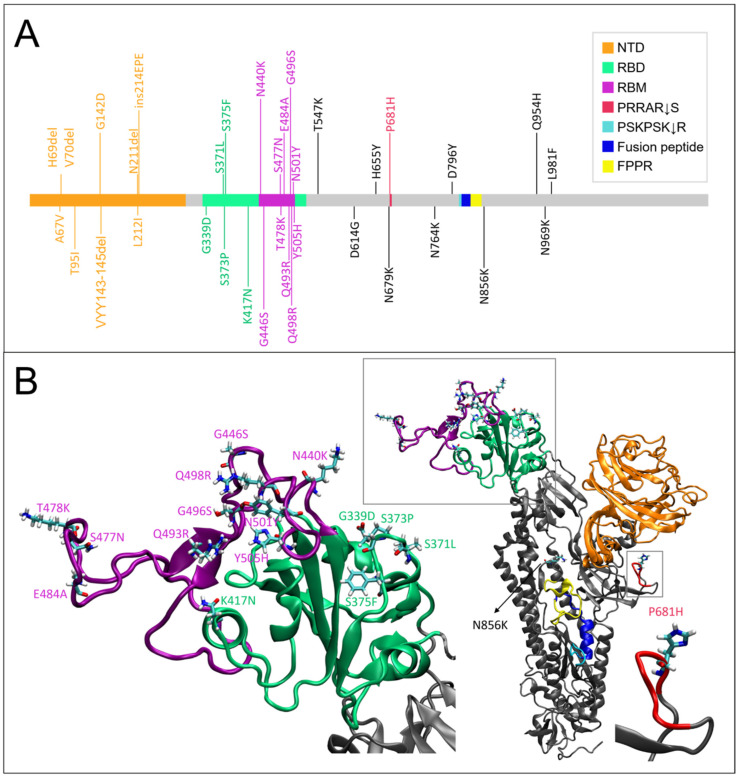
Domain organization and mutations of Omicron variant. (**A**) Graphical scheme of the domains in a spike monomer and Omicron mutations from aa 14 to 1273. (**B**) Cartoon representation of the representative frame (797 ns) of Omicron monomer (mon1) selected by cluster analyses. The WT numbering was maintained. The boxes detail Omicron mutations, respectively, in the RBD domain (in green) and in the cleavage sequence at the S1/S2 boundary (in red).

**Figure 3:**
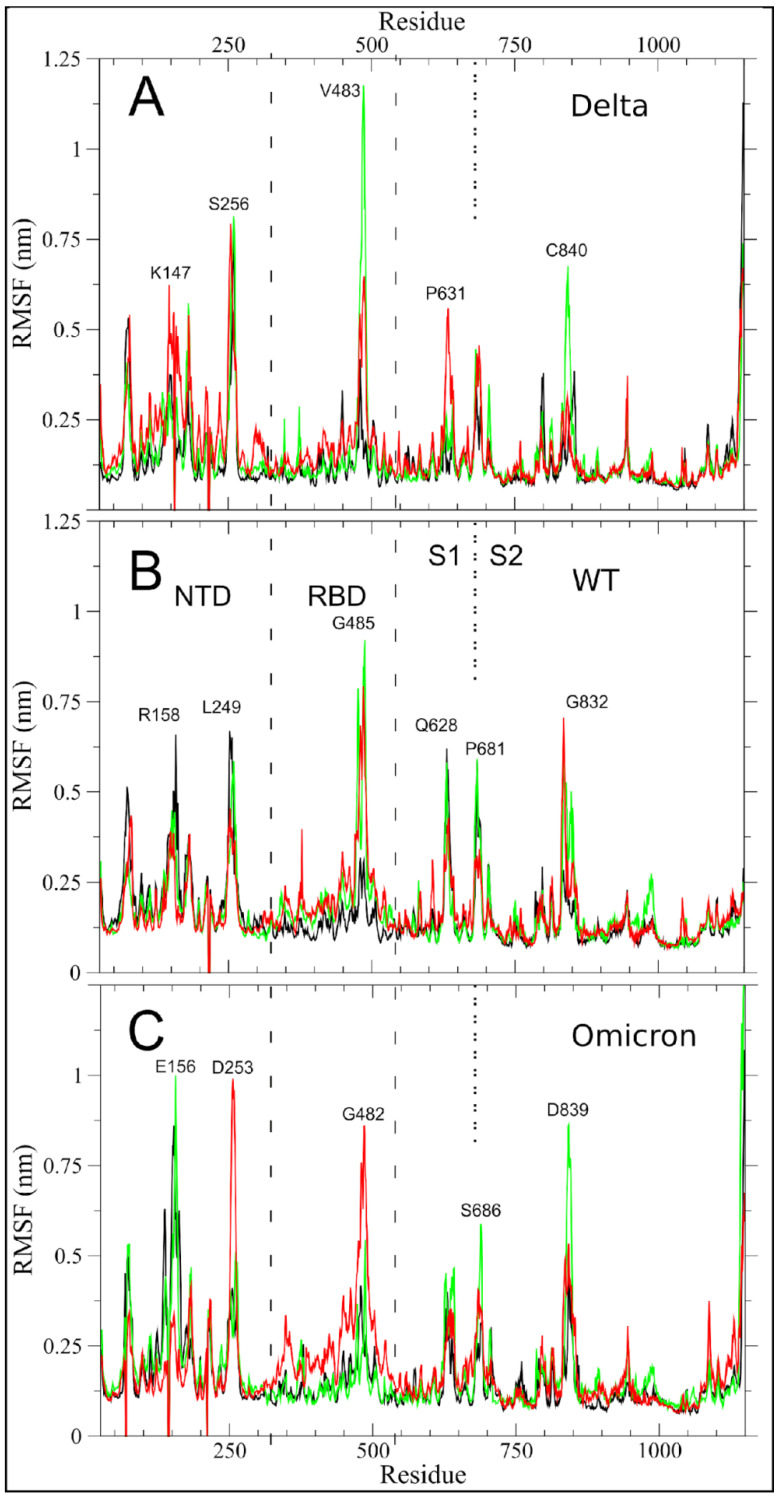
SARS-CoV-2 Delta, WT, and Omicron per-residue RMSF are shown in panels (**A**–**C**), respectively. Spike monomers 1, 2, and 3 are colored in black, red, and green, respectively. Monomer 2 differs from the others by assuming the up conformation (see M&M). The dashed lines indicate the NTD and RBD regions. The dotted line at residue 681 highlights the S1/S2 boundary. The residue numbers of fluctuation peaks are reported as in WT equivalent, not considering the deletion and insertion of mutants.

We first investigated the impact of the mutations that had accumulated in the Delta (Figure 1) and Omicron (Figure 2) variants as compared to WT on the per-residue root mean square fluctuations (RMSF) of S glycoprotein of SARS-CoV-2 in glycosylated form, that were obtained by one-microsecond molecular dynamics (MD) simulations (Figure 3), using an approach that was similar to the work that was done for the structural and dynamic characterization of the Alpha variant (see Borocci and coauthors [25]).

The larger fluctuations in the Delta variant, as in WT, are in the receptor binding motif (RBM) region of the receptor binding domain (RBD), i.e., the region with direct interactions with the Angiotensin-Converting Enzyme 2 (ACE2) human receptor and in the N-terminal domain (NTD). Both regions in Delta, however, have higher maximum values than in WT (comparison of V483 in Delta with G485 in WT; of S256 in Delta with L249 in WT). The monomer in the up conformation (monomer 2), showed an increase in its fluctuation both in NTD and in the P631 region, at the C-term of subdomain 1 (S1), which is one of the two subunits that compose the S protein (see Figure 1A). The first region, with its fluctuation peak in K147, is proximal to the Delta mutations EF156–157del and R158G. The R158 is one of the two most fluctuating residues in the NTD of WT. Therefore, the deletion of residues 156–157 and the mutation in G158 likely produce the observed local alteration around K147, resulting in an increased fluctuation of a larger region of monomer 2.

The peak of fluctuations in subdomain 2 (S2), that are located in G832 of monomer 2 in WT, was maintained in Delta, but with the highest fluctuation located in residue C840 of monomer 3.

Of note is the slight reduction of fluctuations in the residue P681R, the first residue of the canonical furin-like cleavage site (FCS) PRRAR↓S sequence in Delta as compared to WT.

More differences in RMSF are observed in Omicron as compared to WT, as to be expected by its higher number of mutations. The region with the highest fluctuations is the NTD. Here, the increase in fluctuations is found in the two peaks that were already observed in WT and Delta (comparison of E156/D253 in Omicron with R158/L249 in WT and K147/S256 in Delta). We highlight that the peak in D253 involves the monomer in an up-conformation. The RBD is not the region with higher fluctuations in Omicron, but the motion is more distributed along the whole region and also involves monomer 2. 

The FCS region shows fluctuations that are comparable to the WT (comparison of S686 in Omicron with P681 in WT) even though P681 is mutated to a histidine. A moderate increase in fluctuations is observed in the S2 peak (comparison of D839 in Omicron with G832 in WT and C840 in Delta).

### 2.2. Altered Long-Range Correlated Motions in Delta and Omicron S Glycoprotein

To play its biological role, the S glycoprotein must undergo numerous conformational changes, such as RBD rotation in an up-conformation; host-receptor binding; and proteolytic processing at the S1/S2 and/or S2′ sites, close to the fusion peptide, for virus-cell fusion [27]. The Essential Dynamics (ED) technique [28] is well suited for the investigation of the long-range correlated motions that are functional for the orchestration of these S conformational changes [24]. ED has already proved a useful tool in highlighting long-range correlated motions involving key regions in the Alpha variant of the S glycoprotein [25].

After concatenating the trajectories of the three monomers, we performed the ED analyses for Delta, WT, and Omicron, filtering the new trajectories along the eigenvectors with higher eigenvalues. A great portion of the total protein motion in these molecular variants is described by the two main eigenvectors V1 and V2 (89.1 and 88.5% for Delta and Omicron, respectively, to be compared with 88.8% in WT; see Table S1), while V3 contains less than 2% of the total protein motion.

**Figure 4:**
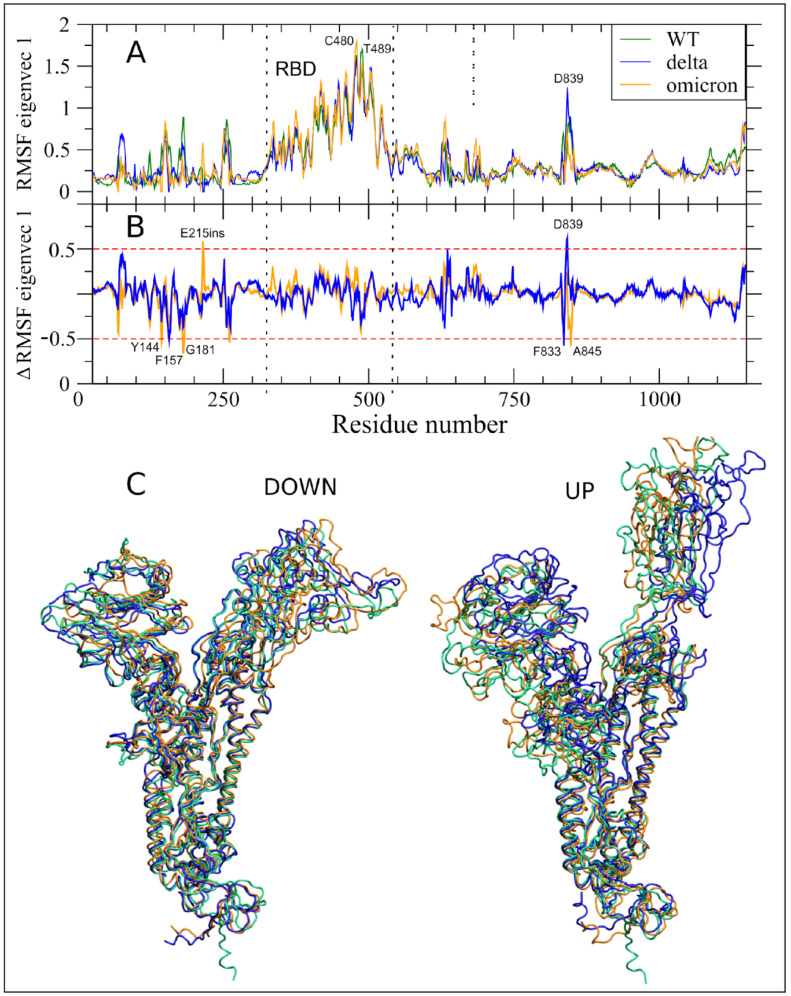
Essential Dynamics V1 analyses. (**A**) RMSF of the S protein filtered trajectory along V1 for WT, Delta, and Omicron are reported in dark green, blue, and orange colors, respectively. Residues corresponding to fluctuation peaks along V1 are indicated. (**B**) Difference between filtered RMSF along V1 between Delta and Omicron as compared to WT are reported in blue and orange colors, respectively. Residues out of the ±0.5 nm threshold (dashed red lines) are indicated. (**C**) Extreme projections of the monomers in down and up conformations along V1 for WT, Delta and Omicron MD trajectories are in green, blue, and orange colors, respectively.

The filtered RMSF along V1 for the three simulations is shown in Figure 4A. The figure shows that the motions along V1 are dominated by the rotation of the RBD between the down and up conformations (peak of fluctuations in C480 of Omicron, P479 in Delta, and T489 in WT), and the correlated motion in the 834–853 aa, named as the fusion peptide proximal region (FPPR) by Cai and coauthors [29]. The correlated motion along V1, connecting RBD and the FPPR region, was already observed in the simulations of S glycoproteins of SARS-CoV-2 ancestors [24] and of the Alpha variant [25], suggesting its relevance in viral pathogenesis and a key role for FPPR has been proposed in the transition between RBD up and down conformations [29].

The similarity of the V1 RMSF of S protein between WT and the two variants can be better evaluated in Figure 4B, where the filtered RMSF difference between these variants and WT is plotted. In both variants, no residues from the RBD region exceed the arbitrary threshold of ±0.5 nm (dashed red line in Figure 4B) where the maximum RMSF is observed (see C480 and T489 in Figure 4A). 

In the Delta variant, F157 in NTD and F833 (i.e., the last residue of the fusion peptide) showed a reduction of RMSF along V1 as compared to the WT. Conversely, the close D839, in the FPPR, showed a large increase in RMSF along V1. The peak that was centered on the residue F157 is due to its deletion in the Delta variant. Therefore, the negative value in Figure 4B corresponds to the fluctuation of F157 along V1 in the WT.

The relative conservation of the motion along V1 of Omicron as compared to WT, notwithstanding its higher number of variants, was unexpected. The negative peak at Y144 was due to the deletion of this residue in the Omicron S glycoprotein. Analogously, the positive peak in the first inserted residue (indicated as E215ins in Figure 4B) is due to the null value in WT. The N-term also shows a negative variation in G181. For Omicron, the only other variation outside of the threshold in a region outside of the N-term is in the FPPR region, as in the case of the Delta variant, where we observe a negative peak in A845.

As in the case of the Alpha variant [25] and other ancestors of SARS-CoV-2 [24], our analysis points to the conservation of the most important correlated motion along V1, that is likely involved in the biological functional role of the S glycoprotein. In this respect, the strong long-range correlated motions between the RBD (the RBM in particular) and the FPPR, close to the peptide fusion region, reinforce this hypothesis. Such long-range communications are needed to orchestrate the different steps of receptor binding and entrance into the host cell.

Moreover, the conservation of the RBD motion along V1 in the three systems, highlighted in the 3D plot of Figure 4C, is indicative that the two RBD mutations in Delta and the 15 in Omicron do not alter the principal up/down motion.

**Figure 5:**
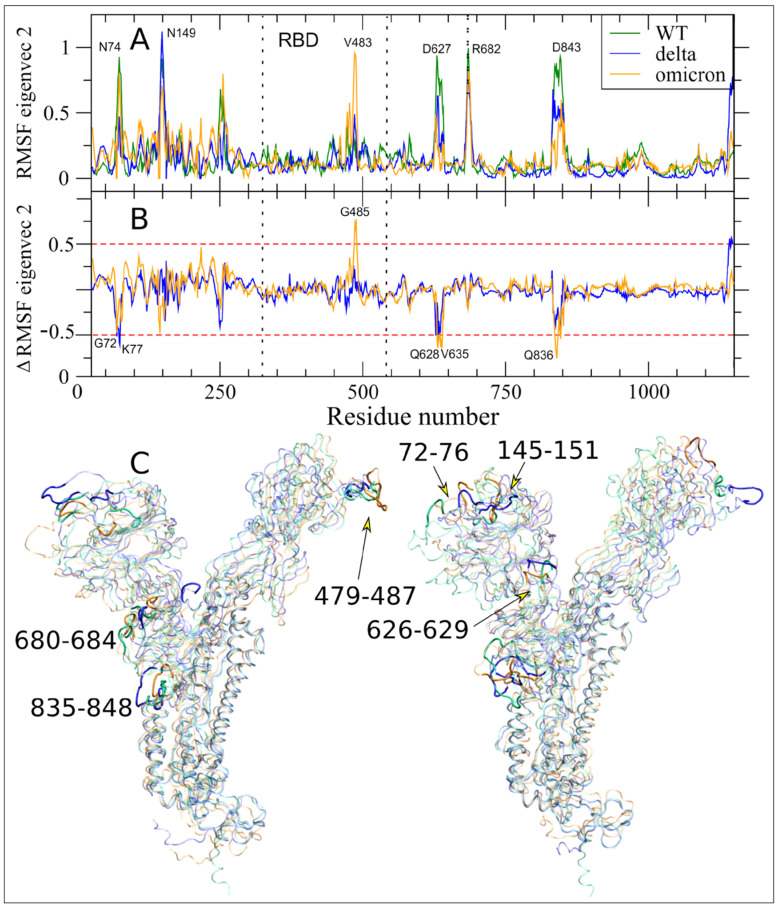
Long-range correlated motions along Essential Dynamics V2. (**A**) RMSF of the S protein filtered trajectory along V2 for WT, Delta, and Omicron variants are reported in dark green, blue, and orange colors, respectively. The residues corresponding to fluctuation peaks along V2 are indicated. (**B**) The difference between the filtered RMSF along V2 between Delta and Omicron as compared to the WT are reported in blue and orange colors, respectively. Residues above the threshold of |±0.5 nm| (dashed red lines) are indicated. (**C**) Extreme projections of the S protein MD trajectory along V2 for WT, Delta, and Omicron variants are in transparent mode and green, blue, and orange colors, respectively. The six regions showing the long-range correlated movements along this ED eigenvectors are in opaque mode and their residue range is reported.

The filtered RMSF of the trajectory along V2 for the three systems (Figure 5) confirms, for the Delta variant, what we already observed in the WT [25], a very limited contribution of RBD to the protein motion along V2 in WT. On the contrary, Omicron showed an important contribution of motion in RBM, with a filtered RMSF peak that was close to 10 Å in V483. This peak is even more intense than what was observed in the Alpha variant (see Figure 4 in Borocci and coauthors [25]). At the same time, the Omicron variant showed reduced motion in other key regions that form the network of long-range communications highlighted by V2, such as the region around N179 in NTD, D627, and FPPR. Other RMSF peaks along V2, finally, have values that are comparable to those of the WT (i.e., the region of N74 and the fusion peptide).

N149 is the only residue with filtered RMSF along V2 that was higher than 10 Å in the Delta variant. This residue is glycosylated, and in WT forms direct interactions with RBD residues, while in the Alpha variant modulates the motion of RBD due to a change in the orientation of its glycans [25], and in Omicron, N149 is close to the VYY143–45del mutation.

Alterations along V2 in the Delta and Omicron variants are highlighted in Figure 5B, which shows the RMSF values subtracted from the WT ones. The studied Delta variant shows a reduction that was greater than 5 Å in two regions: the G72–K77 zone, which is adjacent to the HV69–70del of Omicron and Alpha but not present in Delta; and the 627–634 region. Here, the Omicron variant showed an even greater reduction, with peaks in Q628–V635. Analogously, both variants have a reduction in the FPPR region peaks, but while the Delta variant does not negatively exceed the threshold of -5 Å, the Omicron variant again showed a higher reduction, with the peak in Q836. The only increase in the filtered RMSF along V2 is in the commented RBM of Omicron, with G485, a residue that is involved in ACE2 direct contact, being the residue with the peak value. The residue G485 showed an increase in filtered RMSF V2, and it is greater than 5 Å also in the Alpha variant [25], again suggesting that this ED eigenvector V2, that connects S protein key regions, captures correlated motions that are linked to the observed increased infectivity. The extreme projections of the three molecular systems along V2 are shown in Figure 5C, in transparent mode, with the six peak regions in opaque mode.

### 2.3. Hydrogen Bond Variation in Delta and Omicron Mutated Residues

**Figure 6:**
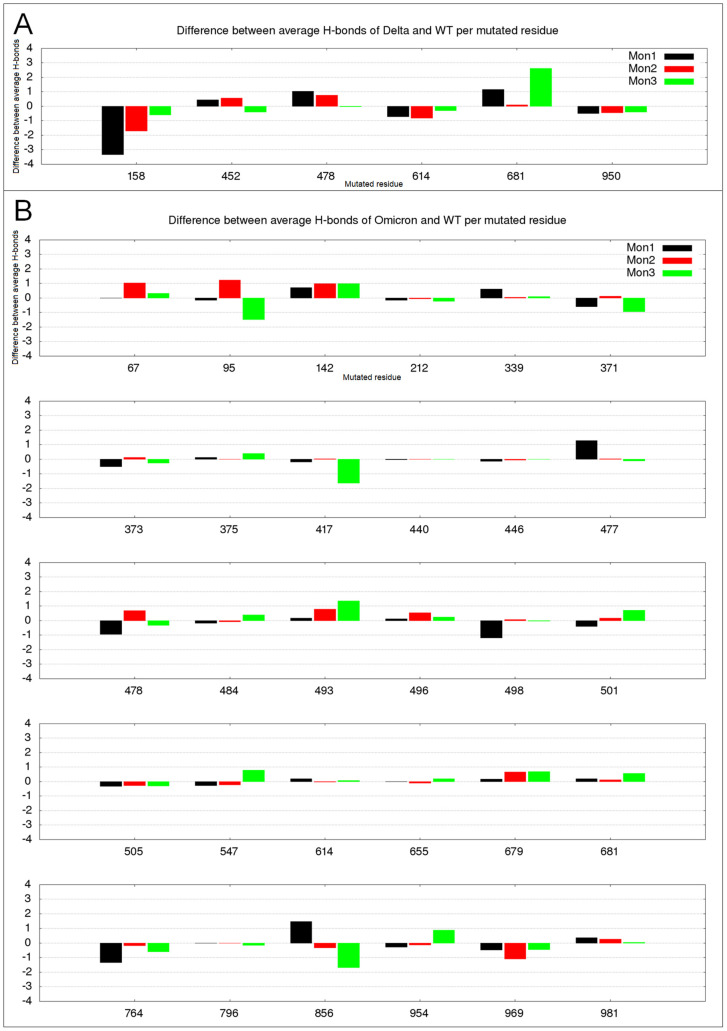
Altered hydrogen bond number in mutated residues. S monomers 1–3 are colored in black, red, and green colors, respectively. (**A**) Differences between the average H-bond numbers in Delta and WT; (**B**) Differences between the average H-bond number in Omicron and WT.

In order to investigate how the local perturbations due to the mutations among the variants produce the observed alteration in the network of long-range correlated motions, we analyzed the changes in the number of direct protein–protein hydrogen bonds in the mutated residues in comparison with the corresponding residue in the WT. The resulting plots for the three monomers are shown in Figure 6. 

Noticeably, the alterations in the Delta variant are the highest, even though the number of mutations is much lower than in Omicron. In particular, R158G and P681R are associated with the loss of more than three hydrogen bonds in monomer 1 and the gain of more than two in monomer 3 (see Figure 6A), respectively. In comparison, the numerous mutations in the Omicron variant never reach a hydrogen bond variation of 2, and rarely exceeded the average value of 1. 

R158 in WT monomer 1, for example, is involved in very stable hydrogen bonds with E156 and more flexible interactions (residence time around 20% of simulation time) with D138, N137, L141, and S162. All these interactions are lost by the Delta mutation in Gly158, and the only hydrogen bond with approximately 25% of the residence time is formed between the main chain of G158 and E154, in monomer 1 and 3. On the contrary, the Delta mutation of P681R produces a very stable hydrogen bond in monomer 1 (84% of residence time) with E661 and more flexible interactions with Q675. On the other hand, this mutation seems to have less influence on the interactions of this residue when the monomer is in the up configuration.

Looking at the interactions that are lost by mutants for deleted residues or gained by Omicron for its insertion, the general trend is maintained, with an average number of hydrogen bonds lost per deleted residue of 0.77 in Delta vs. 0.47 in Omicron, considering also that this last value includes the highest absolute value, i.e., 2.1 hydrogen bonds lost by N211 in monomer 2 that in WT have stable interactions with F186 and K182. Also, the contribution in terms of gained hydrogen bonds by the three inserted residues in Omicron is not distinguishably high, being 0.48.

Overall, the comparison between Delta and Omicron makes us speculate that the S protein might tolerate a few mutations with high impact or several milder ones, in order to maintain its structural biological role.

### 2.4. N-Glycan-RBD Domain Interaction in Delta and Omicron Variants

The N-linked glycans of SARS-CoV-2 S protein are essential for the structure and function of the protein. The glycans are fundamental for the correct folding of the protein, shield the epitopes from the antibody recognition and modulate the structure and dynamics of the S protein [30]. MD simulations [25,31,32], and experimental data [31] show that the glycans linked to asparagine residues 165, 234 and 343 deeply influence the dynamics of the opening of S protein with a profound effect on the biological role of this protein on the viral infection.

In order to investigate the effect of mutations in Delta and Omicron variants on the structure and dynamics of S proteins, we analysed the changes induced by mutations on the glycan-glycan and glycan-protein interactions involved in the modulation of the opening of RBD domain. In particular, we analysed the stability of the hydrogen bond network formed by the glycans close to RBD in “up” conformation. The stability of the glycan-glycan and glycan-protein hydrogen bonds was evaluated in terms of the fraction of MD time in which the hydrogen bond is formed with respect to the MD production time.

**Figure 7:**
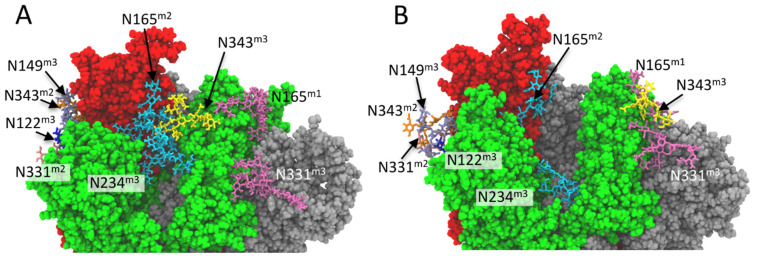
Interactions of N-glycans with RBD domain in up conformation (monomer 2, red color). Monomer colors as in Figure 1C. Enlargement of the region of glycan-glycan and glycan-protein interaction close to RBD of monomer 2 in “up” conformation of the (**A**) Delta(representative frame at 783.8 ns of MD simulation)and (**B**) Omicron variant (representative frame at 893.3 ns of MD simulation), respectively. The amino acids are represented in the CPK model and the glycans (without hydrogen atoms) are represented in licorice. For each residue, the monomer to which they belong is reported as superscript.

In Delta and Omicron variants, as well as in WT, the glycan at N165 (FA2G2S1) of monomer 3 makes stable hydrogen bonds with the amino acid of the RBD domain of monomer 2 in up conformation (Figure 7, Tables S2–S4).

In the Omicron variant, the glycan at N165 interacts also with the RBD domain of the monomer 1 for more than 82% of the MD production time. Specifically, the glycan at N165 makes stable hydrogen bonds with Y498 (24.5% of residence time), G499 (59.1% of residence time), G501 (10.1% of residence time), and H502 (85.1% of residence time).

The glycan that is linked to N234 of the monomer 3 (Man9) does not interact with the RBD domain of monomer 2 in the WT, Delta, and Omicron proteins. In the Delta variant, this glycan makes hydrogen bonds with the amino acids of RBD domain of monomer 3 (intermonomer hydrogen bonds) during all the MD production time (99.9% of residence time). In the Omicron variant, the glycan at N234 forms hydrogen bonds not only with the RBD domain of monomer 3 (69% of the MD production time) but also with the central helix of the monomer 1 (97.7% of residence time). Specifically, the glycan at N234 makes stable hydrogen bonds with S746 (80.7%), E748 (100%) and with T747, N751, N978, F981, N985, and K986 for more than 35% of the MD production time (Table S4).

The glycan that is linked to N343 of monomer 3 (FA2) plays a fundamental role in the mechanism of opening the RBD domain of SARS-CoV2 S protein [33]. In our MD simulation of WT, the glycan at N343 of the monomer 3 (FA2) is mainly oriented toward the RBD domain of monomer 2 (up conformation) and interacts stably with the glycans at N165 and N234 of the monomer 3 with a residence time of 69.5% and 62.3% respectively (Table S5). This stable network of hydrogen bonds between the glycans at N165, N234, and N343contributes to stabilizing the up conformation of monomer 2 and modulates the dynamics of the opening of RBD domain.

In the Delta variant, the interactions between glycan at N343 of the monomer 3 and the glycans at N165 and N234 of the same monomer persist (Figure 7A) even if we observed a reduction in the frequency of glycan–glycan hydrogen bonds with respect to the WT (Table S5). As in the case of the WT, the network of hydrogen bonds that are formed by the glycans at N165, N234, and N343 extends on the top of the deep cavity between the three monomers (Figure 7A) and contributes to modulating the dynamics of the protein.

In the case of the Omicron variant, the glycan at N343 of the monomer 3 does not interact with the glycan N165 and N234 of the same monomer (Table S5). In this variant, the glycan at N343 shows a different orientation with respect to theWT and Delta variants pointing far away from RBD domain in up conformation (Figure 7B) and making stable hydrogen bonds with the glycan at N165 of monomer 1 (FA2G2S2) and the glycan at N331 of monomer 3 (FA3G3S1) with a residence time of 98.6% and 87.6%, respectively (Table S5). 

As previously reported [25], other glycans are involved in the modulation of the flexibility of RBD domain in up conformation. The glycansat N122 (M5) and N149 (FA2) of monomer 3 make a network of hydrogen bonds with the amino acids of RBD of monomer 2 and the glycanat N331 (FA2) and N343 (FA1) of monomer 2. This network of hydrogen bonds is located on the opposite side with respect to the hydrogen bonds network that is formed by the glycans at N165, N234, and N343 (Figure 7A). 

In the Delta variant both the glycans at N122 and N149 make stable hydrogen bonds with the RBD domain in up conformation for more than 97% of the MD production time (Table S3). The glycan at N122 interacts only with the glycan at N331 of the monomer 2 (about 100% of residence time) whereas the glycan at N149 interacts with the glycan at N331 and N343 of monomer 2 for more than 91% of the MD production time (Table S5). 

In the case of the Omicron variant, the glycansthat are linked to N122 and N149 interact through hydrogen bonds, with the RBD domain of monomer 2 with a residence time of 97.6% and 72.4%, respectively. The glycan that is linked to N122 makes stable hydrogen bonds with the glycan N331 and N343 of monomer 2 (above 91% of residence time) whereas the interaction of the glycan at N149 with the glycan N331 and N343 appears weaker with respect to N122 with a residence time of 18% and 77%, respectively.

### 2.5. Secondary Structure Variation in Delta and Omicron Key Regions

The RBD domain plays a crucial role in the interaction with cellular receptors, leading to viral infection. In particular, internal to the RBD domain is the RBM region, which contains residues that are responsible for direct interaction with the ACE2 receptor. To further investigate the impact of Delta and Omicron mutations on this region, we performed secondary structure analyses for the three monomers in the three molecular systems.

**Figure 8:**
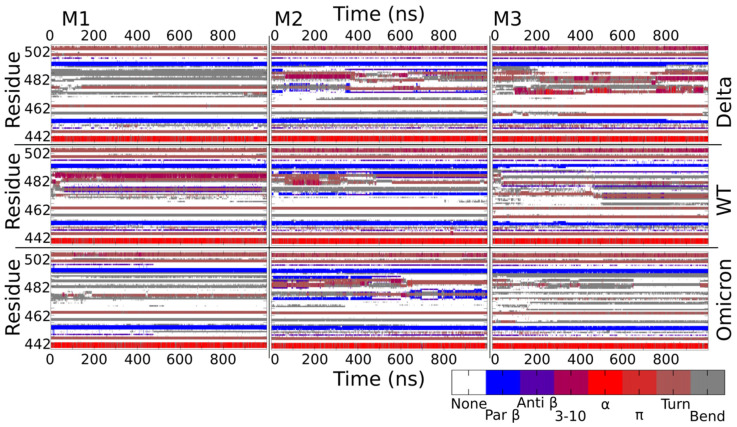
Secondary structure content as a function of the simulation time in M1, M2, and M3 of Delta, WT, and Omicron in the RBM region.

Figure 8 shows that, overall, the secondary structures of the RBM zone are composed of a few structured amino acids that are interspersed with several unstructured loops. It is interesting to note some structural similarities: the alpha-helix (in red) that is centered around residue 441 is followed by a loop and a turn; the two beta-conformations (in blue) that are centered on aa 454 and 494 are zones that are conserved in all monomers of WT and the two analyzed variants. Within these regions, it is evident that the mutations N440K and G446S in Omicron (alpha-helix and turn regions) and L452R in the Delta (beta-conformation that are centered at residue 454) or Q493R and G496S in the Omicron variant (beta-sheet centered at residue 494) do not alter the local secondary structure in any of the monomers, indicating the very high structural stability of these regions.

Among the zones with less conformational stability, we can highlight the regions around aa 477 and 487 that do not have preserved structural patterns either between monomers or between variants. The plasticity of this region was already shown by the MD simulation of the Alpha variant, where we observed the switch of the 479–484 beta-sheet in the up monomer of WT to an alpha-helix conformation [25].

Table S6 summarizes the percentages of secondary structures that were found in the RBM region and corroborates the fact that, on average, more than 60% of this region is composed of loops that intersperse small sets of alpha-helix and beta-sheet that are very well defined and stable that are conserved between the monomers, WT, and variants, providing certain flexibility to the RBM zone that may allow a spatial rearrangement but without losing the structural characteristics of the small regions of secondary structures that make up the region.

## 3. Discussion

In the present study, we have investigated the structural and dynamic effects of the nine mutated residues in the S protein of Delta in comparison to the effects that the 39 mutations in Omicron, ten of which are in the RBM, bring to the S glycoprotein. 

RMSF analysis reveals that the major changes in the Delta S protein, as in WT, are concentrated in the important RBM region that is responsible for the interaction with ACE2 and also in the N-terminal region. Surprisingly, although the Omicron variant has 15 mutations in the RBD region, the average per residue fluctuations are similar to those that were found in theWT, and the biggest differences are in the N-terminal region. Overall, the RMSF (Figure 3) WT peaks are conserved in Delta and Omicron, with relatively small modulation in the absolute values and the residue with the highest value. This result is corroborated by the analyses of essential dynamics, which show: (i) the projection of the ED motion along V1 (Figure 4), where the up/down motion of RBD is the dominant motion, is completely conserved in the three systems; (ii) the projection of the ED motion along V2 (Figure 5), where only three regions in Omicron and one in Delta show variations that are larger than 0.5 Å as compared with the WT.

The hydrogen bond analyses that were performed (Figure 6) point out that, on average, the 30 residues with point mutations in Omicron introduce much smaller changes compared to the six in Delta, which leads us to speculate that more numerous Omicron mutations have a less relevant impact in terms of local H-bonds that are created or broken than Delta mutations.

We demonstrate that glycans play an essential role in modulating the observed S plasticity. A different network of glycan–protein interactions (Figure 7), in fact, explains the increased flexibility of the whole RBD in monomer 2 of Omicron (Figure 3C). These results make us hypothesize whether the increased flexibility of this region can modify the interaction with the ACE2 receptor by facilitating the opening of the RDB domain.

The secondary structure analysis reveals small variation in the RBD region of Delta and Omicron as compared to the WT (Figure 8), where the loops that intersperse small sets of very well-defined secondary structures such as alpha-helixes and beta-sheets, give a general plasticity of some key residues that are responsible for receptor binding. 

Our results suggest that the complex structural changes that allow binding and entering the host cell require great resilience to the general behavior of the S protein. On the other hand, the inherent plasticity of some regions and subtle changes that are induced by the mutations allows for adaptation to the host that might explain the observed extensive evolution and emergence of the SARS-CoV-2 variants.

It is worth noting that coherent structural results highlight the role of specific S regions, i.e., a.a. 624–629, the PRRA cleavage site, and the FPPR region. These regions, in fact, show high correlated motion along V2 in the SARS-CoV-2 ancestor S glycoproteins from bat and pangolin [24], WT, Alpha [25], Delta, and Omicron variants (Figure 5). Moreover, in SARS-CoV-2 as compared to its ancestors, the NTD regions such as the one around N149 and a few residues in the RBM participate in this correlation network. 

Our evidence points out that the structural alterations that are generated by Delta mutations are not very different from those of the Omicron, which makes us believe that, in general, the structure of the S protein can support few more drastic mutations or multiple milder ones, to maintain its biological role. On the other hand, several mutations, even of minor impact from the structural point of view, can modify the glycan network shell, culminating in altering the flexibility of the opening dynamics of the variant and possibly in its interaction with the ACE2 receptor. 

## 4. Materials and Methods 

### 4.1. Modeling of S Glycoproteins Starting Structures

For the modeling of the S glycoproteins, we considered the protocol that was described by Borocci et al. [25]. Briefly for the WT, the initial structure was built with the web tool SWISS-MODEL [34], aligning the reference sequence (NCBI YP_009724390.1, accession number: P0DTC2, SPIKE_SARS2) to the solved cryo_EM structure (PDB entry: 6VYB [35]) to complete the missing residues and restore the FCS sequence to the original SARS-CoV-2 one, that is mutated in the Electron Microscopy experimental structure. For the mutants, the original sequence was changed to correspond with the Omicron (BA.1) and Delta (B.1.617.2) variants, and the following mutations were performed: T19R, EE156–157del, R158G, L452R, T478K, D614G, P681R, and D950Nfor Delta, and A67V, HV69–70del, T95I, G142D, VYY143–145del, L212I, EPE214ins, G339D, S371L, S373P, S375F, K417N, N440K, G446S, S477N, T478K, E484A, Q493R, G496S, Q498R, N501Y, Y505H, T547K, D614G, H655Y, N679K, P681H, N764K, D796Y, N856K, Q954H, N969K, and L981Ffor Omicron. It is worth noticing that the original structure template has a monomer (M2 in our model) in the up conformation, favoring the interaction with the ACE2 receptor.

The three modeled structures were glycosylated following the procedure that was described by Borocci et al. [25] using the GLYCAM-Web tool (www.glycam.org, accessed on 2 December 2021).

### 4.2. Molecular Dynamics Simulations

After the glycosylation of the three models, the S protein was minimized in vacuum with harmonic restraints of 1000 kJ mol^−1^ nm^−2^ on the protein backbone and glycans to avoid close contacts. After this first minimization protocol, the models were added to a cubic box, solvated with water, and neutralized with ions using the genion GROMACS tool. A second minimization step was then performed for the three systems followed by 5 ns of MD simulation by applying positional restraints of 1000 kJ mol^−1^ nm^−2^ to the protein atoms. After, unrestrained MD simulations were carried out with a time step of 2 fs for a length of 1 µs. The P-LINCS [36] algorithm was employed to constrain the H-bonds. The simulations were performed in an NPT ensemble using Parrinello–Rahman barostat [37] to keep the pressure constant at 1 bar and the V-rescale algorithm [38] was employed to maintain the temperature constant at 300 K. The PME [39] algorithm was used to describe the long-range interactions. The proteins, glycans, and water were modeled with Amber14SB [40] force field, GLYCAM06 [41] force field and TIP3P [42], respectively. All the simulations were performed with GROMACS [43] 2020.6 on m100 at the supercomputer center CINECA, Bologna, Italy.

### 4.3. Analyses

The RMSF analyses were performed with the GROMACS rmsf tool and plotted with grace software. To maintain the WT numbering, dummy residues were inserted into the plot. The Essential Dynamics analyses were calculated on c-alpha atoms on the concatenated trajectories for the three monomers by GROMACS covar and anaeig tools, following the protocol that was described by Borocci et al. [25].

Secondary structure data were generated by cpptrajtool [44] and plotted using gnuplot software. For a better understanding, the WT residue numbering was maintained.

The cluster analysis was carried out on the WT, Delta, and Omicron trajectories using the GROMACS cluster tool, according to the algorithm that was described by Daura and coauthors [45]. For all systems, a total of 9000 frames were analyzed and clustered. For WT, the first cluster contains 3592 frames and the centroid (i.e., the frame closer to the middle structure) corresponds to a simulation time of 629,800 ps. For Delta and Omicron, the first cluster contains 4356 and 4923 frames, respectively; and the centroid corresponds to a simulation time of 655.40 and 797.00 ps, respectively.

Hydrogen bond analysis was carried out with VMD [46] and TCL ad-hoc scripts. Hydrogen bonds were defined using the following geometric criteria: we considered the mutated residues and the protein monomers as two functional groups that were linked by hydrogen bond when the distance between heavy atoms (Donor-Acceptor) was less than 0.35 nm and the angle Acceptor-Donor-Hydrogen was smaller than 30°. The plot was obtained from the difference between the H-bonds average number of the mutant and the WT showing the positive bars for the formed bonds and negative bars for the H-bonds that were lost compared to the WT.

MD analyses were carried out on a g100 CINECA supercomputer in the framework of the ELIXIR-IT HPC@CINECA program [47] and on the Tuscia-DIBAF HPC center.

## Data Availability

The data presented in this study are available in article and supplementary material.

## Supplementary Materials

The following supporting information can be downloaded at: https://www.mdpi.com/article/10.3390/ijms23158680/s1.

## Abbreviations

SP	Structural proteins
NSP	Non-structural proteins
SARS-CoV-2	Severe acute respiratory syndrome coronavirus 2
COVID-19	Coronavirus disease 2019
VUM	Variants under monitoring
VOC	Variant of concern
VOI	Variant of interest
WHO	World Health Organization
CDC	Centers for Disease Control and Prevention
WT	Wild-type
RMSF	Root mean square fluctuations
MD	Molecular dynamics
RBM	Receptor binding motif
RBD	Receptor binding domain
ACE2	Angiotensin-converting enzyme 2
NTD	N-terminal domain
FP	Fusion peptide
FCS	Furin-like cleavage site
ED	Essential dynamics
FPPR	Fusion peptide proximal region
