# Supplementary material for: Structural Evolution of Delta (B.1.617.2) and Omicron (BA.1) Spike Glycoproteins
Source: Int J Mol Sci. 2022 Aug 4;23(15):8680. doi: 10.3390/ijms23158680 (PMC9369368; doi:10.3390/ijms23158680)
Supplement: Supplementary file 1 [file ijms-23-08680-s001.zip › ijms-1806565-supplementary.pdf]

Supplementary Materials

Structural Evolution of Delta (B.1.617.2) and Omicron (BA.1) Spike Glycoproteins

Ingrid Guarnetti Prandi[1], Carla Mavian[2,3], Emanuela Giombini[4], Cesare E. M. Gruber[4], Daniele Pietrucci[1,5], Stefano Borocci[1,6], Nabil Abid[7,8], Andrea R. Beccari[9], Carmine Talarico[9,*] and Giovanni Chillemi[1,*]

1 Department for Innovation in Biological, Agro-Food and Forest Systems - DIBAF, University of Tuscia, Via S. Camillo de Lelliss.n.c., 01100 Viterbo, Italy; ingrid.prandi@unitus.it (I.G.P.); d.pietrucci@ibiom.cnr.it (D.P.); borocci@unitus.it (S.B.)

2 Emerging Pathogen Institute, University of Florida, Gainesville, FL 32608, USA; cmavian@ufl.edu

3 Department of Pathology, Immunology and Laboratory Medicine, University of Florida, Gainesville, FL 32610, USA

4 Laboratory of Virology, INMI Lazzaro Spallanzani IRCCS, via Portuense 292, 00149 Roma, Italy; emanuela.giombini@inmi.it (E.G.); cesare.gruber@inmi.it (C.E.M.G.)

5 Institute of Biomembranes, Bioenergetics and Molecular Biotechnologies - IBIOM, CNR, 70126 Bari, Italy

6 Institute for Biological Systems, ISB, CNR, Area della Ricerca di Roma 1, SP35d 9, 00010 Montelibretti, Italy

7 Laboratory of Transmissible Diseases and Biological Active Substances LR99ES27, Faculty of Pharmacy, University of Monastir, Rue Ibn Sina, Monastir 5000, Tunisia; nabilabidbensalem.2014@yahoo.fr

8 High Institute of Biotechnology of Monastir, Department of Molecular and Cellular Biology, University of Monastir, Monastir 5000, Tunisia

9 Dompé Farmaceutici SpA, via Campo di Pile, 67100 L'Aquila, Italy; andrea.beccari@dompe.com

* Correspondence: carmine.talarico@dompe.com (C.T.); gchillemi@unitus.it (G.C.)

Table S1. Percentage of global motion described by the first three Essential Dynamics (ED) eigenvectors (named V1, V2 and V3) in WT, Delta and Omicron.

	**V1**	**V2**	**V3**
**WT**	71.0%	17.8%	1.6%
**Delta**	75.5%	13.6%	1.4%
**Omicron**	74.2%	14.3%	1.9%

Table S2. Frequency (% residence time) of the hydrogen bonds between the glycan linked to N122, N149, N165 and N234 of the monomer 3 and the amino acids of RBDdomains of monomer 2 (up conformation) and monomers 1 and 3.

**Glycan-RBD pairs**	**% residence time**		
	**WT**	**Delta**	**Omicron**
N122-RBD_2 (“up”)	55.0	98.6	97.6
N149-RBD_2(“up”)	96.9	97.7	72.4
N165-RBD_2 (“up”)	97.1	100.0	100.0
N165-RBD_1	0.2	0.5	82.5
N165-RBD_3	79.2	55.5	0.0
N234-RBD_3	99.8	99.9	69.4

Table S3. Frequency (% residence time) of the main hydrogen bonds between the glycan linked to N165, N122 and N149 of monomer 3 and the amino acids of RBD of monomer 2 in up conformation and between the glycan N234 with the RBD domain of monomer 3 in the S protein of Delta variant.

Glycan	Residue	% residence time
N122	Thr 333	6.4
	Asn 334	76.1
	Leu 335	5.5
	Pro 337	0.9
	Glu 340	91.4
N149	Glu 340	92.5
	Asn 343	63.5
	Ala 344	2.0
	Thr 345	5.62
	Asn 370	57.0
	Asn 440	22.2
	Leu 441	1.4
N165	Arg 355	100.0
	Arg 357	21.1
	Asp 427	29.1
	Lys 458	42.8
	Lys 462	38.8
	Phe 464	65.0
	Glu 465	100.0
	Arg 466	100.0
	Asp 467	27.0
	Ile 468	100.0
	Ser 469	36.3
	Glu 471	69.7
N234	Ala 363[1]	23.0
	Asp 364[1]	99.0
	Ser 366[1]	39.3
	Tyr 367[1]	71.4
	Asn 370[1]	18.4
	Ser 371[1]	1.62
	Pro 284[1]	4.0
	Thr 385[1]	18.3
	Asn 388[1]	100.0
	Asp 389[1]	77.0
	Glu 526[1]	6.7
	Pro 527[1]	80.0
	Lys 528[1]	53.1
	Lys 529[1]	27.9

[1]Amino acids of RBD domain of monomer 3

Table S4. Frequency (% residence time) of the main hydrogen bonds between the glycan linked to N165, N122 and N149 of monomer 3 and the amino acids of RBD of monomer 2 in up conformation and between the glycan N234 with the RBD domain of monomer 3 and the central helix of monomer 1 in the S protein of Omicron variant.

Glycan	Residue	% residence time
N122	Thr 333	32.7
	Asn 334	9.7
	Leu 335	52.7
	Asp 339	66.9
	Glu 340	22.4
	Asp 370	9.7
N165	Arg 355	100.0
	Arg 357	37.3
	Asp 427	96.0
	Asp 428	95.6
	Lys 458	24.0
	Asn 460	27.0
	Lys 462	22.4
	Phe 463	67.2
	Arg 466	100.0
	Asp 467	90.4
	Ile 468	68.4
	Glu 471	51.8
N149	Glu 340	1.8
	Asn 343	64.8
	Thr 345	64.7
	Arg 346	19.2
	Asp 352	16.7
	Lys 440	63.3
	Leu 441	52.1
	Ser 443	43.9
	Lys 444	33.0
	Asn 448	11.6
	Asn 450	9.9
N234	Thr 385[1]	31.3
	Lys 386[1]	48.5
	Asn 388[1]	5.0
	Asp 389[1]	41.3
	Lys 528[1]	18.2
	Asp 745[2]	13.6
	Ser 746[2]	80.7
	Thr 747[2]	35.9
	Glu 748[2]	100.0
	Asn 751[2]	32.2
	Asn 978[2]	40.6
	Phe 981[2]	38.0
	Asp 985[2]	41.6
	Lys 986[2]	48.5

[1]Amino acids of RBD domain of monomer 3

[2]Amino acids of monomer 1

Table S5. Frequency (% residence time) of the hydrogen bonds between the glycans N122, N149, N165, N234, N331 and N343 and the amino acids of RBD domain.

Glycanpair	% residence time		
	WT	Delta	Omicron
N165[c]-N234[c]	83.4	39.5	0.0
N165[c]-N343[c]	69.5	61.4	0.0
N234[c]-N343[c]	62.3	27.9	0.0
N343[c]-N165[a]	23.1	40.1	98.6
N343[c]-N331[c]	0.0	0.5	87.6
N122[c]-N331[b]	94.5	99.9	99.8
N122[c]-N343[b]	50.4	0.0	91.0
N149[c]-N331[b]	1.3	73.2	18.6
N149[c]-N343[b]	32.1	98.5	77.3

[a]monomer 1
[b]monomer 2
[c]monomer 3

Table S6. Percentage of residues in specific secondary structure in WT, Delta and Omicron for the RBM region (residues 438-506). Structure corresponds to the sum of the other rows plus Turn.

	WT			Delta			Omicron		
	M1	M2	M3	M1	M2	M3	M1	M2	M3
**Structure**	39.9	39.0	36.2	30.3	36.5	38.8	29.6	39.9	39.0
**β-Sheet**	9.0	13.3	6.4	8.8	9.9	8.1	8.7	9.0	13.3
**β-Bridge**	4.3	2.5	4.5	0.6	3.3	2.8	1.0	4.3	2.5
**α-Helix**	4.6	5.1	4.6	4.2	4.8	4.9	4.3	4.6	5.1
**3-Helix**	4.1	2.0	1.9	0.0	2.9	4.1	0.6	4.1	2.0
